# Supplementary material for: Deterministic processes structure bacterial genetic communities across an urban landscape
Source: Nat Commun. 2019 Jun 14;10:2643. doi: 10.1038/s41467-019-10595-1 (PMC6572833; doi:10.1038/s41467-019-10595-1)
Supplement: Supplementary file 1 — Supplementary Information [file 41467_2019_10595_MOESM1_ESM.pdf]

Supplementary Information

To accompany the manuscript "Deterministic Processes Structure Bacterial Genetic Communities across an Urban Landscape" by Hassell et al.

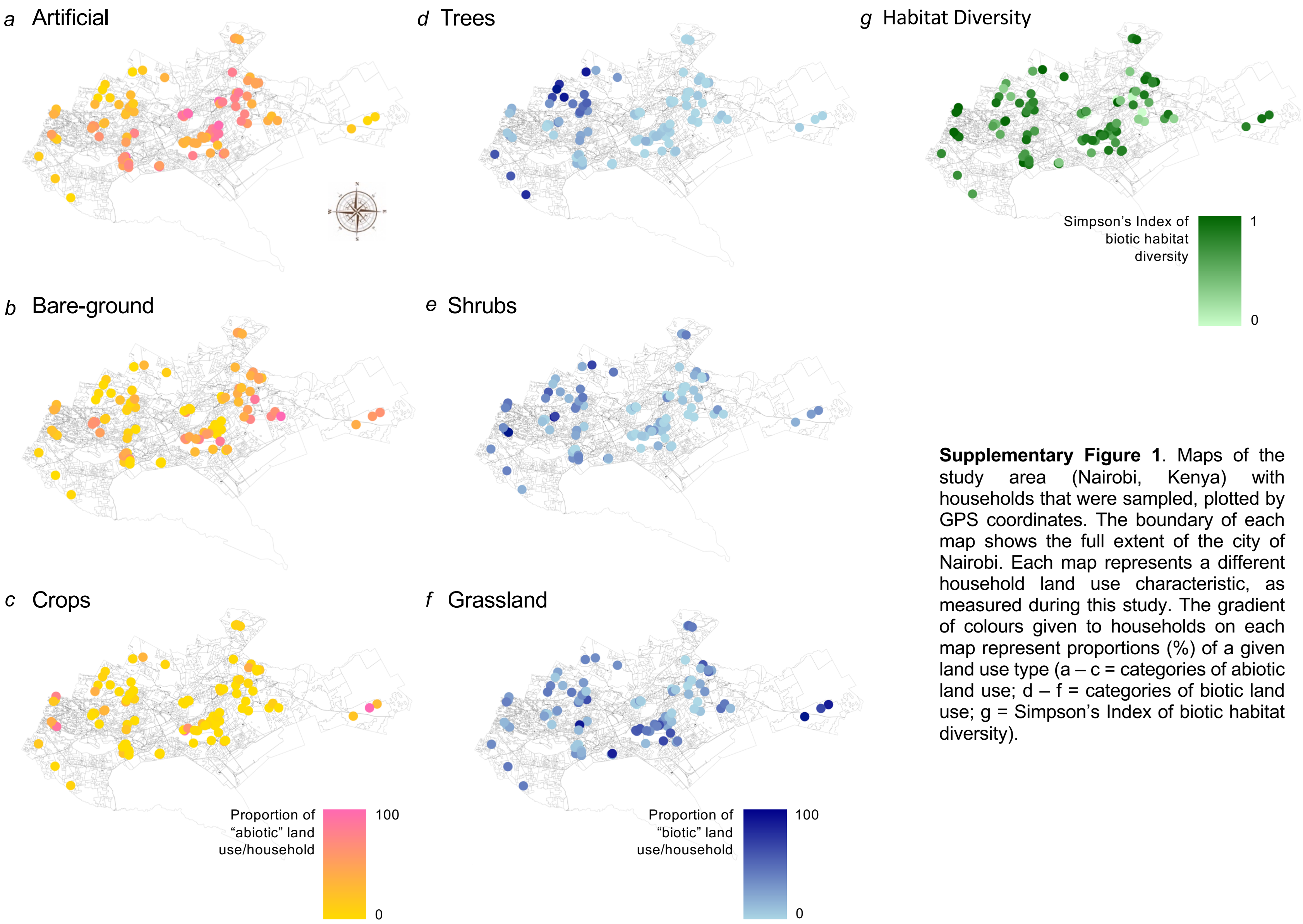

Supplementary Figure 1. Maps of the study area (Nairobi, Kenya) with households that were sampled, plotted by GPS coordinates. The boundary of each map shows the full extent of the city of Nairobi. Each map represents a different household land use characteristic, as measured during this study. The gradient of colours given to households on each map represent proportions (%) of a given land use type (a – c = categories of abiotic land use; d – f = categories of biotic land use; g = Simpson's Index of biotic habitat diversity).

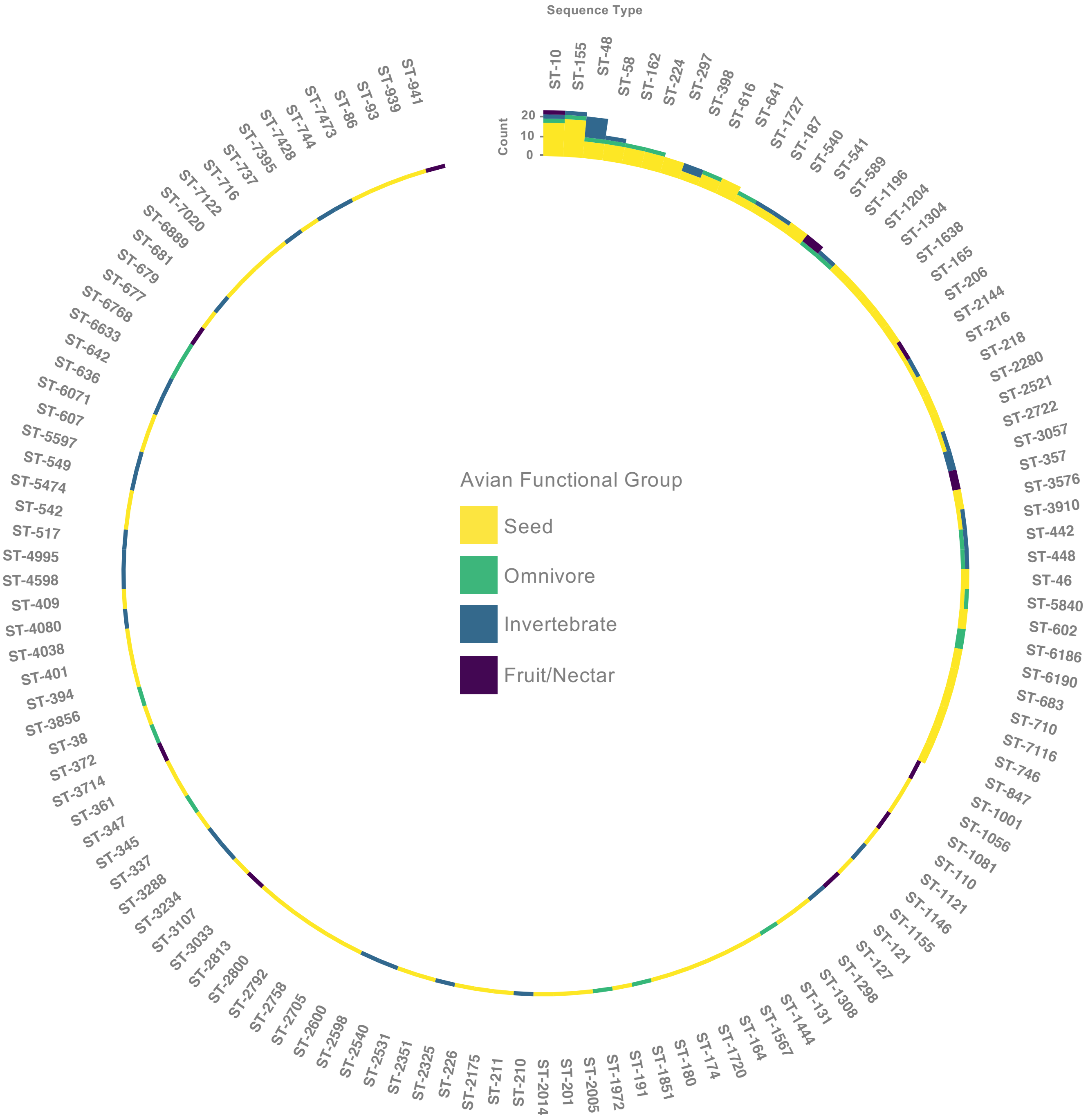

Supplementary Figure 2. Multi-locus sequence types (MLST) of *E. coli* isolates included in this study. Colours indicate avian functional guilds (Yellow = Seed; Green = Omnivore; Blue = Invertebrate; Purple = Fruit/Nectar). 128 unique sequence types were present, and 66% of these (n=85) only appeared in one individual bird.

Characteristics of physical neighbourhood classes identified by IFRA study (adapted from Ledant *et al.* [1])				Urban Zoo Project re-classification		
Tree cover	Defining characteristics	Neighbourhood description (housing type)	Average income	Wealth group	Possible sub-locations	Targeted sub-locations
> 13.5%	Detached housing with intense tree cover	Detached housing on very large plots *(>3000 m^2)*	39,890	1	8	3
		Detached housing on large plots *(400 - 3000 m^2)*	22,462	2	8	4
	Attached and semi-detached housing	Attached housing on medium plots *(<400 m^2)* with important tree cover	22,084	2		
3% < 13.5%	Apartment building	Apartment buildings *with gated space*	22,084	2		
	Attached and semi-detached housing	Higher standing row houses *(plot size > 190 m^2)*	13,352	3	5	3
		Lower standing row houses *(plot size < 190 m^2)*	6,153	4	3	3
<3%	Roof cover >50% tiles	Lower standing *apartment buildings*	6,153	4		
		New areas of dense *single housing* development	3,855	5	9	5
	Roof cover > 40% concrete	High density multi-storey buildings	3,855	5		
3% < 13.5%	Apartment building	Apartment buildings *with open access*	3,855	5		
	Peripheral areas	Peripheral areas with residential component *(mainly residential)*	3,855	5		
		Peripheral areas with rural component *(presence of agriculture)*	2,165	6	24	11
	Collective housing	Community housing *with gated space*	2,165	6		
		Community housing *with open access*	2,165	6		
<3%	Roof cover >85% corrugated iron sheets	New areas of low quality housing *(built-up area <37%)*	2,165	6		
		High density planned low quality housing *(built-up area <37% AND public space >20%)*	2,165	6		
		High density unplanned low quality housing (slums) *(built-up area <37% AND public space <20%)*	1,301	7	13	4

Supplementary Table 1. The seven wealth groups used by the Urban Zoo Project, and the number of sub-locations with a dominant wealth group identified and selected in the Nairobi municipality. Reprinted with permission from Bettridge *et al.* [15].

Land Use Class	Explanation
Water-body (environmental, biotic)	Natural water body (flowing or non-flowing)
Wetland (environmental, biotic)	Open wetland – reeds/rushes
Cropland (anthropogenic, biotic)	Row cops or other herbaceous crops (e.g. maize, coffee etc.)
Trees (environmental, biotic)	Trees (single or multiple), as determined by presence of a clear crown and evidence of shadow cast on ground (includes plantations of trees for commercial purposes [e.g. fruit])
Shrubs (environmental, biotic)	Shrubs, where able to distinguish from grassland and trees
Grassland (environmental, biotic)	Grass, pasture, herbaceous rangeland or bare-ground not serving a human purpose.
Bare ground (anthropogenic, abiotic)	Heavily compacted soil, serving human purpose (e.g. dirt road, playground)
Artificial (anthropogenic, abiotic)	Synthetic, man-made surface or object (including water-bodies) (e.g. tarmac road, cement, roof, swimming pool, water tank)
Rubbish (anthropogenic, abiotic)	Accumulation of human-derived waste

Supplementary Table 2: Land use classifications

Source ID	Genome size	Contigs	n50	Species
SPV065AVN2	5408955	288	141527	ecoli
KIG019AVN5	5390924	157	276611	ecoli
HAR010AVN3	5336375	363	167324	ecoli
DAN033AVN4	5329999	262	150848	ecoli
HAR010AVN5	5323392	262	109285	ecoli
GTN075AVN4	5274763	304	194257	ecoli
KAS061AVN1	5271833	268	176784	ecoli
KAN008AVN6	5269303	201	198291	ecoli
MUT082AVN3	5256944	143	197665	ecoli
KIL042AVN1	5246564	317	97495	ecoli
SPV100AVN4	5227856	175	231214	ecoli
LOR097AVN5	5215757	213	139656	ecoli
LOR097AVN1	5214103	168	183624	ecoli
KTS088AVN11	5179426	511	190484	ecoli
DAN031AVN10	5161950	209	114626	ecoli
KYL026AVN4	5161548	199	122984	ecoli
MAK017AVN8	5159112	197	185273	ecoli
MLK024AVN1	5157088	358	69081	ecoli
EAS044AVN4	5151583	313	114551	ecoli
MGM094AVN2	5150515	400	133269	ecoli
GTN073AVN6	5147195	174	255117	ecoli
MLK024AVN3	5126545	191	140401	ecoli
KAN009AVN8	5125529	168	198399	ecoli
LOR097AVN3	5117083	210	178217	ecoli
KIG019AVN7	5109088	623	186748	ecoli
MIH068AVN7	5108183	367	184123	ecoli
MGM094AVN3	5093486	609	103287	ecoli
DAN031AVN7	5091487	301	154511	ecoli
KIG019AVN9	5087816	132	221964	ecoli
MBO015AVN3	5082100	175	134901	ecoli
MUT083AVN10	5080860	195	192764	ecoli
VIW003AVN2	5071766	198	143988	ecoli
MLK023AVN4	5070155	170	125486	ecoli
KAN007AVN5	5068908	213	139395	ecoli
MSA004AVN3	5063588	240	113624	ecoli
NGN080AVN12	5062565	180	184508	ecoli
GTN073AVN7	5048902	205	141240	ecoli
MUT082AVN1	5035467	230	135165	ecoli
KRR038AVN4	5032806	207	133461	ecoli
GTN073AVN12	5032445	218	135785	ecoli
MGM095AVN2	5031052	323	75616	ecoli
BOM091AVN3	5027847	442	151562	ecoli
KIG020AVN12	5027614	191	174735	ecoli
DAN031AVN12	5023013	453	187358	ecoli
KOR060AVN1	5015932	171	351495	ecoli
EMB085AVN1	5011562	267	144075	ecoli
KIG020AVN3	5007821	119	200439	ecoli
KIG020AVN6	5004286	246	145306	ecoli
KYL025AVN4	5001876	156	210403	ecoli
GTN073AVN3	4994568	119	341414	ecoli
KIL042AVN7	4992808	162	176854	ecoli
GWK076AVN1	4991061	291	71025	ecoli
MSA004AVN2	4989644	174	133197	ecoli
KWG071AVN1	4981960	219	133076	ecoli
MWK057AVN10	4981544	426	150468	ecoli
MWK057AVN14	4981150	860	170042	ecoli
KAS061AVN3	4977994	225	165513	ecoli
KTS089AVN1	4975778	508	154803	ecoli
UTH029AVN3	4975390	162	156833	ecoli

Source ID	Genome size	Contigs	n50	Species
STC047AVN3	4970506	307	162567	ecoli
SPV064AVN6	4968680	261	286902	ecoli
MAK017AVN7	4968036	183	153418	ecoli
KHW050AVN2	4960727	151	211574	ecoli
MIH068AVN2	4958444	168	162476	ecoli
GWK076AVN3	4956745	111	169741	ecoli
DAN031AVN3	4954994	647	225083	ecoli
KIG021AVN3	4950624	227	332563	ecoli
DAN031AVN6	4947179	199	112627	ecoli
HAR012AVN5	4944370	240	84310	ecoli
KIG021AVN2	4943098	377	211086	ecoli
MIH068AVN3	4942891	187	166966	ecoli
KAS061AVN7	4941181	734	13743	ecoli
MBO015AVN1	4939562	173	134351	ecoli
VIW003AVN1	4939198	185	173855	ecoli
GWK076AVN7	4934203	282	93920	ecoli
MAK018AVN9	4931153	130	224563	ecoli
KYL027AVN2	4930819	119	432935	ecoli
VIW002AVN2	4929360	503	182534	ecoli
MAK018AVN6	4928801	133	185318	ecoli
EAS043AVN2	4927391	122	148907	ecoli
MGM095AVN3	4924018	212	159201	ecoli
KRR037AVN6	4921911	210	159160	ecoli
STC046AVN1	4913956	163	152545	ecoli
NGN080AVN9	4913210	159	131810	ecoli
UTH030AVN1	4912702	124	249293	ecoli
MLK024AVN4	4912634	132	212114	ecoli
EMB085AVN5	4910221	95	297053	ecoli
KHW051AVN5	4904037	522	123679	ecoli
STC046AVN3	4901738	276	132527	ecoli
GTN075AVN1	4901145	146	192873	ecoli
KTS090AVN4	4899992	159	185677	ecoli
MGM096AVN1	4896429	1204	88684	ecoli
LUM053AVN3	4895689	605	113693	ecoli
GWK076AVN4	4886125	595	192998	ecoli
KHW050AVN11	4883542	323	172698	ecoli
KAS061AVN4	4882992	262	274510	ecoli
KHW050AVN10	4881533	177	281644	ecoli
KIL041AVN7	4879419	250	191729	ecoli
KYL025AVN2	4879324	177	148683	ecoli
MUT084AVN2	4873414	70	508111	ecoli
GTN075AVN2	4872136	233	140511	ecoli
KIG021AVN5	4871504	153	192576	ecoli
KTS089AVN8	4870729	147	154803	ecoli
KTS088AVN8	4868419	160	104079	ecoli
MBO013AVN4	4868123	202	142600	ecoli
MLK023AVN3	4867965	258	69811	ecoli
GTN073AVN5	4867182	93	235248	ecoli
UTH028AVN10	4866628	168	185567	ecoli
KIG020AVN11	4866058	248	66845	ecoli
SPV100AVN5	4859614	150	232554	ecoli
MUT083AVN4	4858267	180	177299	ecoli
SPV100AVN8	4849457	229	75804	ecoli
GTN073AVN4	4842289	266	185195	ecoli
KYL025AVN1	4841797	140	252669	ecoli
VIW002AVN1	4841244	138	148815	ecoli
KAS061AVN5	4839417	121	265664	ecoli
VIW002AVN3	4839022	142	160944	ecoli
MBO013AVN3	4838682	185	156013	ecoli
MUT084AVN3	4836557	193	247198	ecoli

Supplementary Table 3. Table showing the QC and assembly metrics for the final 241 *E. coli* isolates included in this study.

Source ID	Genome size	Contigs	n50	Species
MLK024AVN7	4835976	362	129794	ecoli
KIL041AVN1	4834919	145	182735	ecoli
LOR098AVN1	4834842	194	117921	ecoli
MIH067AVN7	4830729	106	210527	ecoli
MBO015AVN7	4828898	209	117778	ecoli
MAK018AVN2	4828685	208	131126	ecoli
KIG021AVN7	4823699	124	256820	ecoli
MSA006AVN1	4823692	177	153271	ecoli
KIG019AVN3	4819577	201	216750	ecoli
MWK057AVN12	4817047	676	103746	ecoli
KIG021AVN6	4804893	305	88690	ecoli
KYL026AVN7	4803020	210	100718	ecoli
UTH028AVN7	4802455	146	106222	ecoli
MSA005AVN2	4802104	193	154600	ecoli
UTH028AVN4	4798907	128	193074	ecoli
KHW050AVN4	4798768	334	148372	ecoli
KTS088AVN10	4795894	135	260928	ecoli
GTN074AVN2	4795273	184	160629	ecoli
EMB085AVN6	4794142	133	130028	ecoli
MBO013AVN5	4793434	98	234294	ecoli
MGM096AVN4	4792682	233	184917	ecoli
VIW002AVN5	4790597	257	155829	ecoli
LUM054AVN8	4790288	150	232230	ecoli
MAK016AVN1	4788127	149	162147	ecoli
HAR012AVN7	4787975	631	106755	ecoli
HAR010AVN1	4787750	186	163419	ecoli
HAR012AVN8	4785382	114	190384	ecoli
EMB085AVN8	4785209	201	66302	ecoli
GTN074AVN4	4784491	127	210400	ecoli
KIG019AVN6	4784288	74	414140	ecoli
MWK057AVN11	4780916	322	161178	ecoli
GTN073AVN9	4780136	224	200895	ecoli
MIH067AVN4	4778016	270	146113	ecoli
MSA005AVN4	4777043	98	154710	ecoli
KIG021AVN10	4774754	160	152334	ecoli
VIW002AVN7	4773089	185	154505	ecoli
NGN080AVN10	4770909	108	197585	ecoli
NGN079AVN3	4770087	82	185467	ecoli
BOM091AVN2	4770031	208	157486	ecoli
KYL025AVN3	4763262	162	279491	ecoli
KTS088AVN14	4762344	84	210930	ecoli
SPV100AVN6	4759109	156	154795	ecoli
NGN079AVN2	4757174	178	187657	ecoli
SPV064AVN13	4753836	271	259439	ecoli
GWK076AVN6	4748367	186	85629	ecoli
SPV100AVN2	4747865	212	137713	ecoli
MAK017AVN2	4743446	193	158731	ecoli
UTH030AVN2	4743329	120	203866	ecoli
UMJ035AVN4	4742001	232	72098	ecoli
NGN080AVN13	4736830	213	122233	ecoli
KRR037AVN8	4735454	113	187241	ecoli
DAN032AVN2	4732065	178	147603	ecoli
HAR011AVN1	4731553	539	74957	ecoli
KAN008AVN2	4730971	185	101104	ecoli
EMB085AVN9	4730342	92	214000	ecoli
GTN074AVN3	4729027	197	107104	ecoli
KHW051AVN6	4728761	119	213152	ecoli
MIH067AVN1	4727512	112	218080	ecoli
MBO013AVN1	4727129	221	67781	ecoli
SPV100AVN7	4724768	181	109347	ecoli

Source ID	Genome size	Contigs	n50	Species
MSA006AVN3	4724145	185	112476	ecoli
KHW050AVN7	4718280	261	147589	ecoli
GTN075AVN3	4715737	190	185196	ecoli
GTN073AVN8	4714717	89	274067	ecoli
MBO013AVN2	4707737	176	111688	ecoli
DAN031AVN11	4707612	221	69273	ecoli
EMB085AVN7	4706280	82	378193	ecoli
EMB087AVN2	4705547	252	110554	ecoli
MAK017AVN9	4704904	144	159823	ecoli
KTS088AVN1	4703650	272	154554	ecoli
KAS061AVN8	4698953	204	59618	ecoli
KWG071AVN2	4698281	236	92117	ecoli
KYL026AVN5	4697237	193	180605	ecoli
MWK056AVN3	4697203	510	101675	ecoli
KIL042AVN8	4696740	134	185259	ecoli
MAK018AVN7	4694629	191	88670	ecoli
HAR012AVN4	4693653	202	92076	ecoli
GTN073AVN11	4691571	122	194756	ecoli
GWK076AVN5	4690589	132	216345	ecoli
HAR012AVN9	4686409	116	208276	ecoli
MIH068AVN6	4684802	148	185367	ecoli
EAS044AVN1	4682992	135	199136	ecoli
KHW051AVN1	4679647	298	209566	ecoli
KHW050AVN1	4679066	136	173755	ecoli
KYL026AVN9	4676714	130	177414	ecoli
VIW002AVN4	4676249	192	86036	ecoli
MAK017AVN5	4676133	51	541224	ecoli
MWK057AVN5	4675820	195	195340	ecoli
EMB085AVN3	4672718	316	61209	ecoli
SPV064AVN11	4667202	178	184573	ecoli
KHW051AVN2	4663271	183	71942	ecoli
MUT083AVN7	4661619	69	422212	ecoli
GTN073AVN2	4661089	193	129605	ecoli
KOR059AVN2	4659266	247	90363	ecoli
MAK018AVN4	4657853	104	198234	ecoli
KIL042AVN4	4656303	232	97761	ecoli
MAK018AVN8	4656244	207	87567	ecoli
MIH067AVN5	4653409	187	70894	ecoli
LUM052AVN3	4644498	380	90773	ecoli
STC048AVN1	4642666	146	160630	ecoli
GTN074AVN1	4641452	67	365234	ecoli
LOR098AVN7	4635995	148	111071	ecoli
EAS044AVN9	4632970	142	186984	ecoli
MGM095AVN4	4631952	143	182712	ecoli
DAN031AVN2	4628964	107	185258	ecoli
KYL026AVN11	4622437	294	96934	ecoli
MUT083AVN11	4620154	101	185679	ecoli
UTH028AVN2	4617383	84	196226	ecoli
UTH028AVN8	4609418	131	197097	ecoli
EAS044AVN3	4607314	165	94109	ecoli
NGN079AVN5	4596650	340	73284	ecoli
SPV064AVN9	4586767	173	92511	ecoli
KIG019AVN4	4584909	53	265194	ecoli
BOM091AVN1	4584733	116	123945	ecoli
KAN009AVN1	4573792	158	110311	ecoli
KIG021AVN8	4572765	223	91284	ecoli
KYL027AVN6	4570093	177	98576	ecoli
MLK024AVN5	4552885	162	96809	ecoli
KHW051AVN4	4525753	265	99458	ecoli
MAK016AVN2	4517195	123	143589	ecoli
MAK017AVN3	4509790	217	84450	ecoli
HAR010AVN7	4490062	163	106257	ecoli

Supplementary Table 3 contd.
