# Supplementary material for: Deterministic processes structure bacterial genetic communities across an urban landscape
Source: Nat Commun. 2019 Jun 14;10:2643. doi: 10.1038/s41467-019-10595-1 (PMC6572833; doi:10.1038/s41467-019-10595-1)
Supplement: Supplementary file 3 — Reporting Summary [file 41467_2019_10595_MOESM3_ESM.pdf]

nature research

Corresponding author(s): DBPR NCOMMS-18-28797

Reporting Summary

Nature Research wishes to improve the reproducibility of the work that we publish. This form provides structure for consistency and transparency in reporting. For further information on Nature Research policies, see Authors & Referees and the Editorial Policy Checklist.

Statistical parameters

When statistical analyses are reported, confirm that the following items are present in the relevant location (e.g. figure legend, table legend, main text, or Methods section).

n/a	Confirmed	
☐	☒	The exact sample size (*n*) for each experimental group/condition, given as a discrete number and unit of measurement
☐	☒	An indication of whether measurements were taken from distinct samples or whether the same sample was measured repeatedly
☐	☒	The statistical test(s) used AND whether they are one- or two-sided<br>*Only common tests should be described solely by name; describe more complex techniques in the Methods section.*
☐	☒	A description of all covariates tested
☐	☒	A description of any assumptions or corrections, such as tests of normality and adjustment for multiple comparisons
☐	☒	A full description of the statistics including central tendency (e.g. means) or other basic estimates (e.g. regression coefficient) AND variation (e.g. standard deviation) or associated estimates of uncertainty (e.g. confidence intervals)
☐	☒	For null hypothesis testing, the test statistic (e.g. *F*, *t*, *r*) with confidence intervals, effect sizes, degrees of freedom and *P* value noted<br>*Give P values as exact values whenever suitable.*
☒	☐	For Bayesian analysis, information on the choice of priors and Markov chain Monte Carlo settings
☐	☒	For hierarchical and complex designs, identification of the appropriate level for tests and full reporting of outcomes
☐	☒	Estimates of effect sizes (e.g. Cohen's *d*, Pearson's *r*), indicating how they were calculated
☐	☒	Clearly defined error bars<br>*State explicitly what error bars represent (e.g. SD, SE, CI)*

Our web collection on statistics for biologists may be useful.

Software and code

Policy information about availability of computer code

Data collection	Field data were collected using the open source software Open Data Kit (ODK) Collect.
Data analysis	All data analysis was conducted using open source packages in the statistical computing platform R v.3.3.2.

For manuscripts utilizing custom algorithms or software that are central to the research but not yet described in published literature, software must be made available to editors/reviewers upon request. We strongly encourage code deposition in a community repository (e.g. GitHub). See the Nature Research guidelines for submitting code & software for further information.

Data

Policy information about availability of data

All manuscripts must include a data availability statement. This statement should provide the following information, where applicable:

- Accession codes, unique identifiers, or web links for publicly available datasets
- A list of figures that have associated raw data
- A description of any restrictions on data availability

Data are available via an open access repository held by the University of Liverpool (http://datacat.liverpool.ac.uk/id/eprint/526).

Field-specific reporting

Please select the best fit for your research. If you are not sure, read the appropriate sections before making your selection.

☐ Life sciences ☐ Behavioural & social sciences ☒ Ecological, evolutionary & environmental sciences

For a reference copy of the document with all sections, see nature.com/authors/policies/ReportingSummary-flat.pdf

Ecological, evolutionary & environmental sciences study design

All studies must disclose on these points even when the disclosure is negative.

Study description	The work presented in this paper formed part of the 'UrbanZoo 99 household project', based in Nairobi, Kenya between 2012-2017, which focused on informal livestock keeping practices in urban households as a route of zoonotic disease emergence in humans. The project was designed as a cross-sectional study, utilizing multi-stage cluster sampling by splitting the city into 33 sublocations that were proportionately chosen to represent a gradient of socioeconomic housing types, and thus urbanization across the city. These sublocations represent the first-level of clustering. Within each sublocation, three randomly selected households represented the second level of clustering (which number 99 in total), within which samples of wild birds represent the cluster sampling 'element'.<br><br>Households were selected with the aim of maximizing the spatial distribution and diversity of livestock keeping practices across Nairobi, and were chosen to capture three main criteria: socio-economic diversity, population distribution and livestock keeping practices. Geospatial mapping data, generated as part of a technical report produced by Institut Français de Recherche en Afrique (IFRA), was used to identify 17 classes of residential neighborhood in Nairobi based on physical landscape attributes, which were subsequently verified by 817 household questionnaires. Each of the 17 classes of neighborhood were then ranked by average income and reduced into seven wealth groups. Administrative sublocations were mapped onto each wealth group, identifying a total of 70 possible sublocations, for which dominant wealth groups were calculated by extracting the proportion of population belonging to each neighborhood class within the sublocation boundaries. A total of 33 sublocations were selected to be included in the study, with the number of sublocations belonging to each wealth group chosen proportionately to the population density and the variety of neighborhood classes in each of the seven wealth groups. Final selection of individual sublocations was aimed at maximizing areas with high livestock densities, whilst ensuring coverage of other neighborhood classes and geographical spread. For each sublocation, three geographical points were selected at random within the dominant housing type. The order in which sublocations were visited was randomized. Local officials assisted in the recruitment of a household closest to each geographical point, to obtain two livestock keeping and one non-livestock keeping household per sublocation (a total of 99 households, 66 of which kept livestock). Households had to meet strict inclusion criteria of keeping either large ruminants (cattle), large monogastrics (pigs), small ruminants (goats/sheep), small monogastrics (poultry/rabbits), or no livestock species. To ensure an equal sample of both cattle and pig-keeping households, the combination of livestock keeping households represented in each sublocation was randomized, and had to consist of either large ruminant and small monogastric, or large monogastric and small ruminant species. For sublocations in which households keeping large ruminant or large monogastric species were absent, a replacement household keeping either small monogastic or small ruminant species was recruited.
Research sample	57 species of wild bird (of both sexes) were sampled in 99 households across Nairobi, Kenya. This population sample is broadly representative of wild birds present at urban wildlife-livestock-human interfaces across a rapidly developing city. Birds were chosen as wildlife hosts in this urban study system, since diverse avian communities distribute widely across urban landscapes, demonstrating epidemiological and ecological responses to land-use change, and interacting closely with livestock and humans.
Sampling strategy	Due to the nature of the study design, and the fact that we were dealing with population genetic measures for which it is very difficult to predict biological significance in advance, we were unable to generate meaningful statistical power calculations or sample sizes to answer the genetic questions posed in this study. As such, birds were opportunistically trapped in household compounds using mist nets. Due to large variation in the size of household compounds, trapping effort (i.e. number of mist nets placed per trapping session) was maintained such that it was proportional to the size of the household compound, and thus standardized across households.
Data collection	Trained veterinarians collected feces or a cloacal swab from each wild bird, before it was released unharmed. The diversity (species richness) of avian communities within each household compound was characterized by an ornithologist from the National Museums of Kenya. A nominated member of each household completed a questionnaire, detailing i) livestock ownership, management, sourcing, sales and antimicrobial use, and ii) household composition and socio-economic data. All field data was recorded using Open Data Kit (ODK) Collect software, on electronic tablets, and uploaded to databases held on servers at the International Livestock Research Institute (ILRI). Household land use was characterized by drawing the boundary of each household compound in ArcMap, including a 30m buffer around the perimeter of each compound to represent the landscape surrounding it. Visual classification of land-use types within the compound and buffer area were conducted at 1:500 scale on a 1m resolution ESRI World Imagery satellite-image available in ArcGIS 10.5 (ESRI). Within the boundary, the areas of nine different land-use types were visually identified and sketched as polygons; water-body, wetland, crops, mature trees, shrubs, grassland, bare ground, artificial ground and rubbish. The total area of classified land-use types at each site were calculated and expressed as proportions.<br><br>All swabs and fresh faecal samples were placed in Amies transport media and transported on ice to one of two laboratories (Kenya Medical Research Institute (KEMRI) or University of Nairobi (UoN)). Samples were enriched in buffered peptone water for 24 hours, and plated onto eosin methylene blue agar (EMBA). Plates were incubated for 24 hours at 37oC, after which five colonies were selected from each EMBA plate. After a further sub-culture on EMBA to purify the isolates, the pure isolates were sub-cultured on Müller-Hinton (MH) agar and archived at -80oC in cryovials containing Soy broth supplemented with 15% glycerol.

A single colony was picked from each original sample (referred to as an 'isolate') and biochemical tests (triple sugar iron agar, Simmon's citrate agar, and motility-indole-lysine media) were run for identification as E. coli. DNA was extracted from bacterial isolates using commercial kits (Purelink® Genomic DNA Mini Kit, Invitrogen, Life Technologies, Carlsbad, California) and transported under licence to The Wellcome Trust Centre for Human Genetics, Oxford, UK. Whole genome sequencing (WGS) was carried out at the Wellcome Trust Centre for Human Genetics on the Illumina HiSeq 2500 platform. 150 base-pair paired-end reads were generated and short-read WGS data were pre-processed using an automated protocol developed by the Modernising Medical Microbiology Oxford (MMM) Group to: (i) perform standard quality control checks using fastQC (https://www.bioinformatics.babraham.ac.uk/projects/fastqc/) with default settings; (ii) trim reads to remove remnant adaptor sequences using bbduk (parameters: minoverlap=12, k=19, mink=12, hdist=1, ktrim=r) and (iii) perform a Kraken speciation analysis against with an in-house database of bacterial reads downloaded from the NCBI sequence read archive (www.ncbi.nlm.nih.gov/sra/), with an automated step for removal of contaminant (non-bacterial) reads. De novo assembly was performed using SPAdes v3.642 (parameters: --careful, -t 1, --phred-offset 33). The assemblies were run through the batch upload mode of the Centre for Genetic Epidemiology web interface hosted by the Technical University of Denmark (https://cge.cbs.dtu.dk/services/cge/) which performs speciation analysis, multilocus sequence typing (MLST), detection of resistance genes and detection of virulence genes. The threshold of AMR gene detection was set to 90% identity and 60% coverage, as this is shown to be the optimal threshold for this method. A 60% coverage threshold was used to ensure that AMR genes spread over two contigs, and/or located on the edge of the contig, were not missed. Virulence genes were identified using VirulenceFinder with 90% minimum match and 60% minimum length. Samples deemed as non-E. coli on the basis of the speciation analysis with kmerFinder in the Centre for Genetic Epidemiology pipeline were excluded from further analysis. Potentially mixed E. coli samples were identified as those with an unusually large assembly size (greater than 6 megabases (Mb)) and were removed from the dataset.

Timing and spatial scale

Field data was collected between September 2015 and September 2016. Triplets of households with each sublocation were sampled within the same week. All field data collection for each household was conducted on the same day.

Data exclusions

Isolates deemed not to be E. coli, on the basis of biochemical testing or whole genome sequencing, were removed from the dataset.

Reproducibility

Trapping effort (i.e. number of mist nets placed per trapping session) for wild birds was maintained such that it was proportional to the size of the household compound, and thus standardized across households. Characterization of avian communities within each household was standardized (being conducted by the same ornithologist, and based on audio-visual identification over a 20-minute period spent walking transects of each household compound). Field samples were sent to one of two laboratories for microbial culture, and all efforts were undertaken to ensure that this did not introduce bias into the study. Protocols were standardized between laboratories, and field samples were sent in duplicate to each laboratory as a quality control measure.

Randomization

Geographical points used to select households within each sublocation were distributed at random. The combination of livestock keeping households represented in each sublocation was randomized, and had to consist of either large ruminant and small monogastric, or large monogastric and small ruminant species. The order in which sublocations were visited for data collection between September 2015 and September 2016 was randomized. One purified E. coli isolate per original sample grown was selected at random.

Blinding

N/A

Did the study involve field work? ☒ Yes ☐ No

Field work, collection and transport

Field conditions

Field work was conducted over the course of one year, and as such precipitation and temperature varied over the course of the study. Nairobi spans Afromontane and Savannah biomes, and as such natural habitat varied markedly between households.

Location

Sampling was conducted in households across the city of Nairobi, Kenya.

Access and import/export

The collection of data adhered to the legal requirements of the International Livestock Research Institute (ILRI), and country in which the research was conducted. Wild birds were trapped under approval of an ILRI Institutional Animal Care and Use Protocol (2015.12), and questionnaire data was collected under ILRI Institutional Research Ethics Committee approval (2015-09). Permission to conduct research on birds was granted by the National Museums of Kenya (NMK/ZLG/TRN/6.1, 2015.03). E. coli DNA was exported from Kenya to The Wellcome Trust Centre for Human Genetics, Oxford, UK under a Kenyan Ministry of Agriculture license RES/POL/VOL XXIV/72.

Disturbance

Wild birds were released unharmed, at the same location and into the same habitat, from which they were trapped.

Reporting for specific materials, systems and methods

Materials & experimental systems

n/a	Involved in the study
☒	☐ Unique biological materials
☒	☐ Antibodies
☒	☐ Eukaryotic cell lines
☒	☐ Palaeontology
☐	☒ Animals and other organisms
☐	☒ Human research participants

Methods

n/a	Involved in the study
☒	☐ ChIP-seq
☒	☐ Flow cytometry
☒	☐ MRI-based neuroimaging

Animals and other organisms

Policy information about studies involving animals; ARRIVE guidelines recommended for reporting animal research

Laboratory animals

The study did not involve laboratory animals

Wild animals

547 wild birds (of both sexes), representing 57 avian species, were trapped in this study. Birds were trapped using mist nets, positioned around each household compound, and livestock keeping areas. Birds were removed from nets, manually restrained and sampled at the site of capture, and released unharmed.

Field-collected samples

All swabs and fresh faecal samples were placed in Amies transport media and transported on ice to one of two laboratories (Kenya Medical Research Institute (KEMRI) or University of Nairobi (UoN)). Samples were enriched in buffered peptone water for 24 hours, and plated onto eosin methylene blue agar (EMBA). Plates were incubated for 24 hours at 37oC, after which five colonies were selected from each EMBA plate. After a further sub-culture on EMBA to purify the isolates, the pure isolates were sub-cultured on Müller-Hinton (MH) agar and archived at -80oC in cryovials containing Soy broth supplemented with 15% glycerol.

A single colony was picked from each original sample (referred to as an 'isolate') and biochemical tests (triple sugar iron agar, Simmon's citrate agar, and motility-indole-lysine media) were run for identification as E. coli. DNA was extracted from bacterial isolates using commercial kits (Purelink® Genomic DNA Mini Kit, Invitrogen, Life Technologies, Carlsbad, California) and transported under licence to The Wellcome Trust Centre for Human Genetics, Oxford, UK. Whole genome sequencing (WGS) was carried out at the Wellcome Trust Centre for Human Genetics on the Illumina HiSeq 2500 platform. 150 base-pair paired-end reads were generated and short-read WGS data were pre-processed using an automated protocol developed by the Modernising Medical Microbiology Oxford (MMM) Group to: (i) perform standard quality control checks using fastQC (https://www.bioinformatics.babraham.ac.uk/projects/fastqc/) with default settings; (ii) trim reads to remove remnant adaptor sequences using bbduk (parameters: minoverlap=12, k=19, mink=12, hdist=1, ktrim=r) and (iii) perform a Kraken speciation analysis against with an in-house database of bacterial reads downloaded from the NCBI sequence read archive (www.ncbi.nlm.nih.gov/sra/), with an automated step for removal of contaminant (non-bacterial) reads. De novo assembly was performed using SPAdes v3.642 (parameters: --careful, -t 1, --phred-offset 33). The assemblies were run through the batch upload mode of the Centre for Genetic Epidemiology web interface hosted by the Technical University of Denmark (https://cge.cbs.dtu.dk/services/cge/) which performs speciation analysis, multilocus sequence typing (MLST), detection of resistance genes and detection of virulence genes. The threshold of AMR gene detection was set to 90% identity and 60% coverage, as this is shown to be the optimal threshold for this method. A 60% coverage threshold was used to ensure that AMR genes spread over two contigs, and/or located on the edge of the contig, were not missed. Virulence genes were identified using VirulenceFinder with 90% minimum match and 60% minimum length. Samples deemed as non-E. coli on the basis of the speciation analysis with kmerFinder in the Centre for Genetic Epidemiology pipeline were excluded from further analysis. Potentially mixed E. coli samples were identified as those with an unusually large assembly size (greater than 6 megabases (Mb)) and were removed from the dataset.

Human research participants

Policy information about studies involving human research participants

Population characteristics

Household questionnaire data was used to record the number of people residing and/or working in each household (subsequently used to generate an estimate of human density per household), and composite measures of household wealth and 'ruralness'. These indices were calculated based on methods used to create the Demographic and Health Surveys (DHS) wealth index, which is derived from a Principal Component Analysis (PCA) of easily measurable households assets (such as access to water, construction materials and ownership of livestock). A modification was made to the original set of household assets included in the DHS index to better capture household variation in Nairobi.

Recruitment

Local chiefs and elders for each sublocation in Nairobi assisted in recruitment, which was generally carried out several days before the visit to the households. In the presence of these local officials, the random geographical points were identified on the ground, and the nearest households were identified that met inclusion criteria, to obtain a triplet of two livestock-keeping and one non-livestock-keeping households in each sublocation.

Human members of a household were defined as those who either slept on the premises, or (in order to include staff) spent at least 8 hours a day on the plot most days and interacted regularly with the core household in a way likely to facilitate sharing of pathogens, such as sharing food prepared on the premises, regularly handling food, animals, animal manure or contact with human excrement. Other families who lived on the plot (such as tenants) but who had separate cooking facilities and did not contact or share livestock or livestock products belonging to the core household were excluded.

Once households had been identified and consented to participate, appointments were made for visits by the study teams. Most field staff were Kenyan nationals, fluent in both Kiswahili and English, and participants could opt to take the questionnaires in either of these languages.
